# Waste-derived carbon nanomaterials for electrochemical applications: toward a circular and sustainable future

**DOI:** 10.1039/d5ra08357e

**Published:** 2026-01-05

**Authors:** Shruthi Venkataraman, Ashok K. Sundramoorthy

**Affiliations:** a Centre for Nano-Biosensors, Department of Prosthodontics and Materials Science, Saveetha Dental College and Hospitals, Saveetha Institute of Medical and Technical Sciences, Saveetha University India ashok.sundramoorthy@gmail.com

## Abstract

The unparalleled global surge in waste generation necessitates a revolutionary shift toward the circular economy, simultaneously addressing resource depletion and environmental remediation. This review highlights the critical role of various waste materials, such as plastic waste, industrial waste, and agricultural waste, used for the synthesis of nanomaterials like carbon nanotubes, graphene, and carbon quantum dots, as versatile, high-performance precursors for advanced technology. Specifically, we demonstrate how precise tuning of synthesis enables predictable control over the final material's structural, morphological, and surface properties. These characteristics, such as high conductivity and specific surface area, are essential for their remarkable performance in key electrochemical domains. This review emphasizes the impact of waste-derived carbon nanomaterials on electrochemical sensors, where they enhance detection sensitivity and selectivity to achieve ultra-low limits across environmental and biomedical applications. Finally, we addressed the pressing challenges of the field, including the necessity for scalable, energy-efficient synthesis protocols and toxicity assessment, defining a clear future roadmap to integrate these sustainable materials into industrial-scale sensing and energy conversion systems.

## Abstract 2

This review underscored the profound potential of upcycling diverse waste streams ranging from plastic and electronic waste to agricultural biomass into high-value CNTs, graphene, and CQDs and their use in electrochemical sensors.

## Graphical abstract

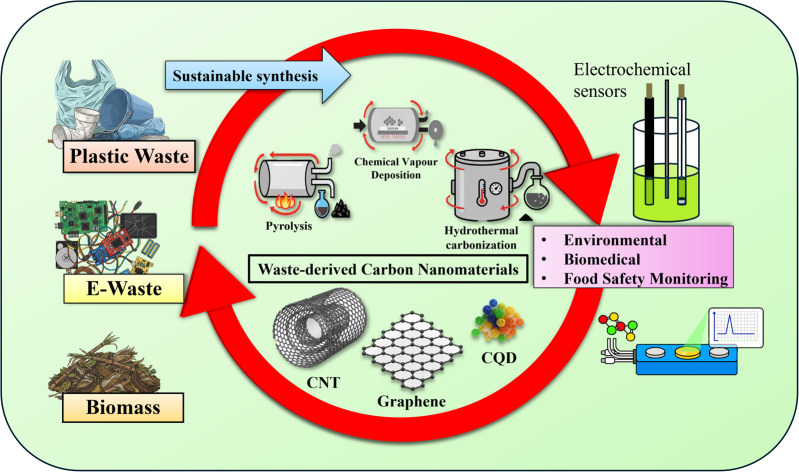

## 1. Introduction

The unparalleled increase in the population and the development of the global economy have led to a multiple-fold increase in waste generation in the past few years. The world generates approximately 2.01–2.24 billion tons of municipal solid waste annually, reported as of 2022, with at least 33% not being managed properly. The volume of waste averages 0.74 kilograms per person per day globally, but this may, however, vary from person to person depending on the region and income level.^1–3^ Looking forward, it is estimated that the global waste to grow to 3.40 billion tons by 2050. Daily per capita waste generation is said to increase by 19 percent by 2050 in high-income countries, whereas an increase by more than 3 times is projected from low-income countries. One of the largest single waste streams, accounting for 30–40% of total solid waste, is the construction and demolition waste, predicted to reach 2.2 billion tons by 2025.^4–7^ However, while generated in smaller volumes, municipal solid waste represents the most monitored waste stream, where food and green waste almost comprise 44% of the entire municipal waste generated.^8–10^ Electronic waste has reached a record of 62 million tons in 2022, representing an 82% increase from 2010. This type of waste is anticipated to increase to 82 million tons by 2030.^11–15^ Plastic waste also poses a critical challenge, with global production reaching 415 million tons in 2023 and waste generation expected to reach 460 million tons by 2025.^16–20^ With extensive awareness campaigns, less than 9–10% of this waste is recycled globally, while 8 million tons enter the oceans annually. Food waste contributes 8–10% of annual global greenhouse gas emissions, nearly five times the total emissions from the aviation sector.^21–23^

**Fig. 1:**
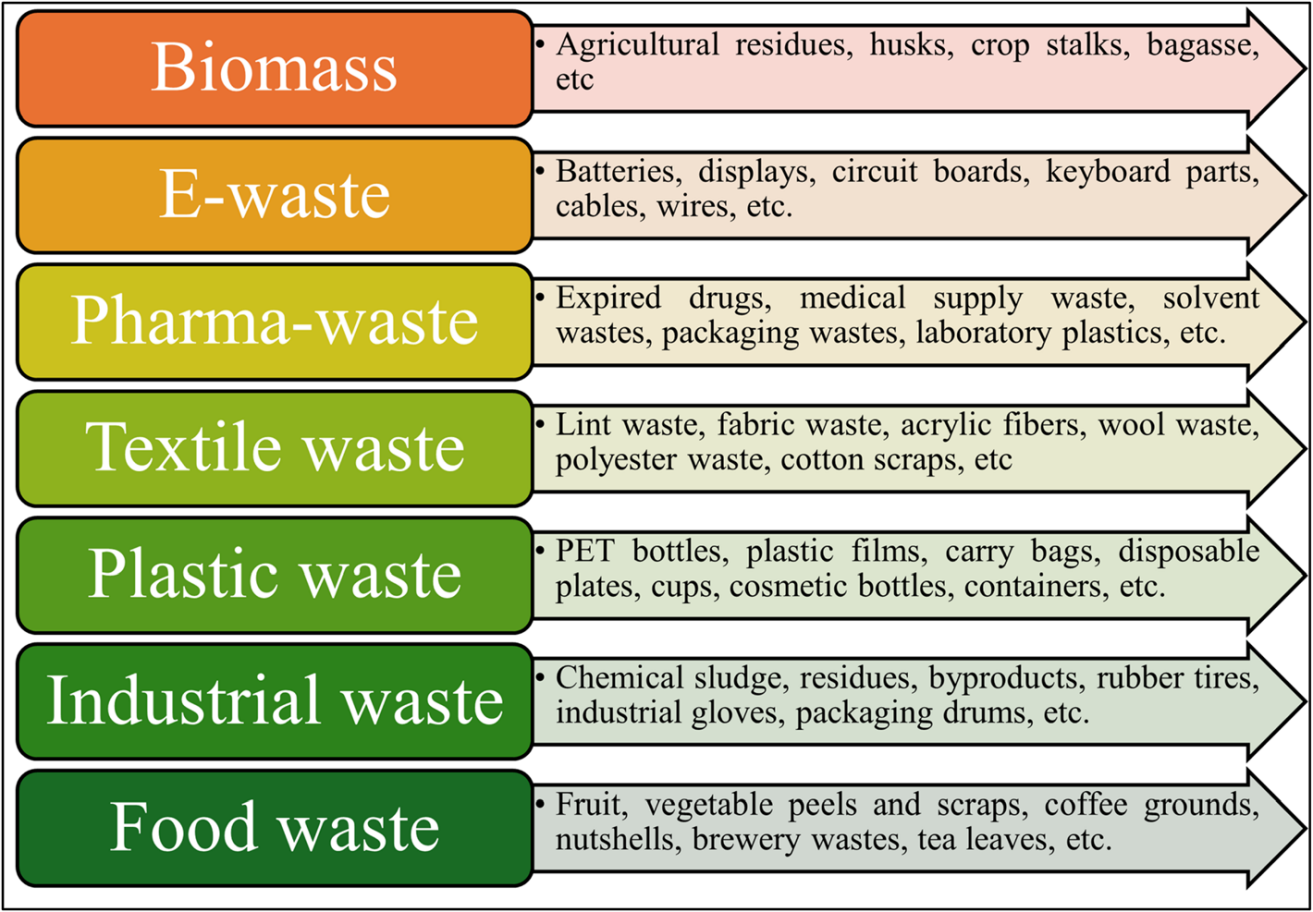
Categories of waste materials commonly used as sustainable precursors for carbon nanomaterial synthesis. These diverse waste streams offer valuable carbon sources for producing a wide range of nanostructured materials. This figure was prepared using Microsoft PowerPoint.

To deal with this constant increase, these wastes must be managed efficiently. Current global waste management reveals that about 35% of waste globally goes to landfills, 33% is openly dumped.^24^ In contrast, 93% of the waste ends up in open dumps, especially in low-income countries. According to the World Bank, about 11% of waste produced globally is incinerated,^24–26^ and the United Nations Environmental Programme (UNEP) reports that 17% of plastic waste is being incinerated.^27^ This increased waste generation globally calls for stringent waste management strategies.^28^ Biomedical waste, E-waste, and industrial waste are a few of the wastes that require proper deactivation and disposal, as they are loaded with toxins that could be harmful when mixed with water bodies, and also get contaminated in the wind.^29^ It has always been difficult to manage this enormous amount of waste generation, so most of them gets ended up in landfills.^30^ Strategies like the circular economy must be taken into account in these cases. Unlike the traditional linear economy of take, use, and dispose culture, the circular economy urges us to reuse, repair, and recycle.^31,32^ To address the pressing challenge of waste accumulation, there is a need for innovative scientific approaches that can simultaneously manage waste utilization and support sustainable development through the use of advanced technologies.^33,34^ In this context, nanoscience and nanotechnology have emerged as powerful tools, offering promising strategies for tackling global issues such as solid waste management.^35^ Recent progress highlights how research efforts have successfully combined efficient low-cost synthesis of waste-derived carbon nanomaterials (WDCNMs), such as carbon nanotubes (CNTs), graphene, and carbon quantum dots (CQDs), for diverse industrial applications.^36^ These carbon nanomaterials (CNMs) derived from waste sources exhibit exceptional properties like high aspect ratio, remarkable electrical conductivity, high mechanical strength, and excellent thermal stability.^37^Fig. 1 provides a schematic representation of various waste sources that provide immense potential as a precursor for carbon nanomaterial synthesis. This review discusses the recent global data on waste generation, management and the conversion of diverse waste streams into high-performance carbon nanomaterials like CNT, graphene, and CQDs. It also addresses the circular economy crisis and enables the development of highly sensitive and sustainable electrochemical sensing technologies for environmental and biomedical applications. The literature search was conducted across various databases, specifically Scopus, PubMed, and Web of Science, to ensure the coverage of recent advancements. The research articles were collected in the period of January 2020 to December 2025. The main keywords were used, such as ‘waste-derived carbon nanomaterials’, ‘electrochemical sensors’, ‘carbon nanotubes’, ‘graphene’, and ‘carbon quantum dots’ to refine the search and identify relevant articles. Although many reviews discussed waste-derived carbon materials, most of them focus on either synthesis or broad applications. In contrast, this review brings the aspect of involving the chemistry of different waste streams with the synthesis conditions that shape the final yield of carbon nanomaterials. It also compares how variations in temperature, pressure, doping, and reaction environment influence the resulting morphology. This targeted approach offers a practical pathway from waste precursor to sensing application within the framework of a circular economy.

## 2. Modern synthesis approaches for carbon nanomaterials

Carbon nanomaterials (CNs) can be synthesized from a wide range of waste materials, where each contributes to enhancing their performance in technological applications.^38,39^ The increasing production of waste materials due to the increasing population and the expansion of the global economy demands an immediate and long-term solution to compensate for the crisis.^40^ To curb this ongoing issue, the best ethical way is to convert this waste into value-added nanomaterials such as CNTs, graphene, and CQDs.^41^ Waste materials generated range from household to agricultural to industrial wastes. Household waste and agricultural waste mostly comprise less toxic waste compared to the large amount generated from the industries.^42^ Carbon nanomaterials being synthesized from any of these waste materials are made possible due to the presence of carbonic skeletons in their fundamental framework.^43^ With this advantage, it is convenient to derive materials in the nanoscale range. The structural morphology of the synthesized nanomaterials also depends on the method used to synthesize them.^44^ Numerous different methods are available to convert waste materials into CNMs, which include pyrolysis, hydrothermal carbonization, chemical vapor deposition (CVD), and physical vapor deposition (PVD).^45–47^ Each of these methods is unique in producing CNMs with different morphologies. However, there are also various parameters to consider, like temperature, pressure, pH, and residence time, and so on, with these methods.^48^

### 2.1. Various synthesis methods of CNTs

Pyrolysis is one of the most relevant technologies when it comes to CNMs synthesis. It uses pyrolytic gases, such as nitrogen, methane, and carbon dioxide, in the absence of oxygen. The temperature used to carry out pyrolysis ranges from 500 to 1000 °C, in tubular furnaces.^49^ This method produces high-quality materials that are more suitable for high-performance applications. Polyurethane (PU) and ethylene-vinyl acetate (EVA) (shoe waste) plastics were recycled into CNTs *via* pyrolysis-CVD over Fe/MgO. PU favored few-walled CNTs with narrow diameters, while EVA produced multi-walled CNTs. By adjusting PU/EVA ratios, CNT size distribution could be tailored. CNTs from PU or PU/EVA mixtures showed excellent performance as electrocatalysts for CO_2_ reduction, achieving 85–95% faradaic efficiency due to enhanced conductivity and surface area.^50^ Catalytic pyrolysis method was carried out with real-world waste plastics (RWWP) as a route to synthesize CNTs. Using thermogravimetric analysis (TGA) and fast-pyrolysis TG/mass spectrometry (Py-TG/MS) analyses, the plastics were identified as polystyrene (PS), polyethylene (PE), polypropylene (PP), and polyethylene terephthalate (PET). Among these, PS, PE, and PP demonstrated super carbon sources, yielding volatile hydrocarbons that supported CNT growth. However, PET produced oxygenated species with poor carbon deposition. A two-stage pyrolysis-catalysis process over Fe@Al_2_O_3_ generated multi-walled CNTs (MWCNTs) with graphitic structure, high purity (up to 93%) with a carbon yield of 32.2 wt%.^51^ Many pyrolysis methods require higher temperatures, often between 600 to 1000 °C, to penetrate the feedstocks and enable carbon deposition. This makes the process energy-intensive and however, with high heating rates, energy input is also increased. In a study, waste PP materials were used for the synthesis of CNTs, and it has been proven that with increased CVD temperature, the quality of the CNT is improved, but the yield is compromised. Comparatively, higher heating rates reduced both the quality and yield of the material produced.^52^

**Fig. 2:**
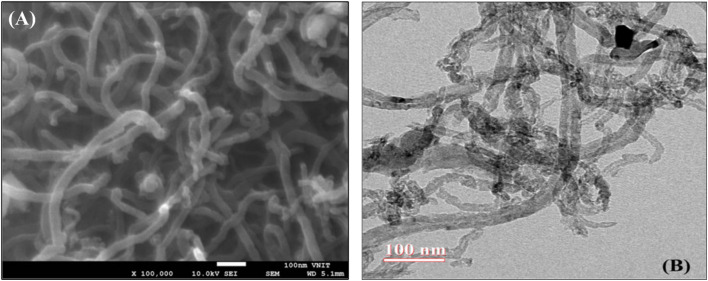
(A) SEM and (B) HRTEM images of MWCNTs synthesized from waste toner powder. Reproduced from ref. 53 with permission from Taylor & Francis Group, LLC, copyright 2019.^53^

Various structural morphologies of carbon nanomaterials can be achieved by employing different techniques and parameters. For instance, a study demonstrated the synthesis of MWCNTs from waste toner powder using the CVD method. Proximate analysis confirmed the toner's high volatile matter content (56.7%) and carbon composition (49.37%), making it a suitable precursor. A Ni_4_Mo_0_._2_MgO_1_ catalyst prepared by the sol–gel method was used, and the parameters were optimized using response surface methodology, achieving a maximum yield of 215% at 950 °C for 8 minutes with 0.4 g of catalyst and 10 g toner. X-ray diffraction confirmed a graphitic structure. Scanning Electron Microscopy (SEM) and TEM, high-resolution transmission electron microscopy (HRTEM) (Fig. 2A & B) revealed uniform MWCNTs with outer diameters of 40–50 nm and inner diameters of 10–20 nm.^53^

CVD offers exceptional control over CNT properties, which is used to tailor nanotube characteristics for specific applications. The method provides precise control over diameter, length, alignment, and even chirality through careful selection of catalysts, substrates, and process conditions.^54^ This method has proven to be useful in the mass production of CNTs.^55^ CVD is used to convert volatile carbon sources to solid non-volatile carbon products using catalysts.^54^ Recent studies show that ash-based secondary waste materials can act as a catalyst source for the growth of nanostructures. Using microwave plasma-enhanced CVD, wastes were used directly without any pre-treatment to grow CNT-rich porous structures. Growth was achieved with or without methane as an added carbon source, indicating that the inherent carbon content of the waste can also contribute to the CNT formation.^56^

Another eco-friendly method to synthesize CNTs from waste is hydrothermal carbonization. Hydrothermal processing uses hot, pressurized water as the reaction medium, where controlled temperature and pressure cause phase changes and chemical transformations.^57^ Under these conditions, key properties of water such as viscosity, ionic product, and thermal conductivity alter in ways that directly influence the reaction pathway.^58^ Unlike conventional approaches that rely on water simply as a solvent, reactant, or catalyst, modern hydrothermal methods use engineered reactors and optimized parameters to convert complex waste feedstocks into fuels, chemicals, or functional carbon materials.^59^ In the subcritical region, water's altered behavior also increases the solubility and reactivity of both polar and non-polar components in biomass.^60^ This environment enables a wide range of reactions like hydrolysis, dehydration, decarboxylation, condensation, and aromatization that collectively drive carbon enrichment and the formation of hydrochar.^61,62^ A study reports the conversion of PET into N-doped carbon nanotubes through a nitric acid-assisted hydrothermal process. During synthesis, intrinsic defects in PET-derived CNTs promote N-doping *via* NH_3_ released from nitric acid, which was confirmed by experimental and theoretical analysis. This approach is more sustainable than CVD methods using acetylene.^63^ Another study focused on using Fe_2_O_3_–Al_2_O_3_ catalysts to convert polyolefin plastic waste into MWCNTs and hydrogen gas. Traditional catalyst synthesis typically occurs at lower temperatures and atmospheric pressure, but this study uses hydrothermal conditions to investigate the role of various precipitating agents, including urea, *N*-methyl urea, and *N*,*N*′-dimethylurea. Subsequently, the study summarized that specific precipitating agents influenced catalyst properties like surface area and reproducibility, which in turn affected the yield and diameter of the resulting MWCNTs. Specifically, the product synthesized with urea exhibited the highest surface area and catalytic activity. This study also concludes that appropriate precipitating agents during hydrothermal synthesis are key to producing highly efficient catalysts for upcycling plastic waste.^64^

### 2.2. Waste to graphene based nanomaterials

**Fig. 3:**
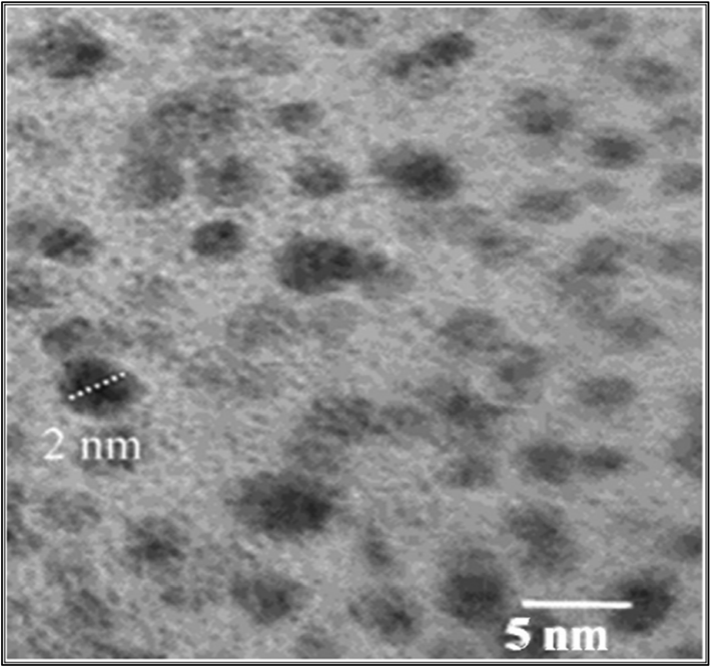
TEM analysis of N-GQDs synthesized from waste resources. Reproduced from ref. 71 with permission from Elsevier B.V., copyright 2021.^71^

Similar to CNTs, other carbon-based nanomaterials can be synthesized using waste materials. These waste materials are mostly composed of a layered carbon structural framework, which makes it possible to synthesize 2D nanomaterials from them. Graphene, one of the well-known carbon materials, is a single layer of graphite and is the thinnest allotropic form of carbon.^65–67^ It also possesses the highest specific surface area, excellent electrical strength, and thermal conductivity due to the presence of C–C sigma covalent linkage.^68,69^ Graphene synthesized from waste materials has also shown these similar qualities. One such study examines the utilization of plastic waste to produce graphene nanosheets. These synthesized nanosheets were then deposited over Cr_2_O_3_, CuO, and NiO nanoparticles as unsupported catalysts. For different catalysts, a different graphene deposition route was used, where a simple two-step pyrolysis method was used to synthesize graphene sheets over metal oxide nanoparticles. The study summarizes that Cr_2_O_3_ produces more graphene than the other two.^70^ Another study reported the synthesis of highly fluorescent nitrogen-doped graphene quantum dots (N-GQDs) from waste *via* microwave treatment, and their morphological characteristics were confirmed through TEM images (Fig. 3).^71^

Another research paper details an evaluation of hydrothermal carbonization as a technique to transform textile-derived waste into valuable carbonaceous products. Using real microfibers collected from clothes washers and dryers, researchers have explored the effect of various temperatures and residence times to optimize the conversion process. It demonstrated that these conditions tailored the product yields, producing either amorphous carbon at 250 °C or high-value nanomaterials like graphene and graphite when processed at 300 °C.^72^ A recent study reports a sustainable, non-toxic route for producing graphene oxide and reduced graphene oxide from waste coconut shells. These shells were converted into graphite through controlled carbonization, subsequently to synthesize GO using modified Hummer's method. The results demonstrate that the coconut-shell-derived GO and rGO exhibit structural quality and functional properties in comparison with the commercial graphite. This study also highlights the potential of this synthesized material in biomedical use, particularly in bone tissue regeneration.^73^

Even though plastic wastes are produced in massive quantities, it is also notable that agricultural and food waste are generated on an everyday basis. While these wastes are biodegradable and can be repurposed for various purposes, most of them are discarded without any effective usage. Discarded dragon fruit peels were used as a natural reducing agent as an alternative to conventional ones. These fruit waste-derived components demonstrated great promise for the graphene oxide reduction.^74^

### 2.3. Waste to CQDs

Concerning 0-dimensional nanomaterials, carbon quantum dots are one of the most common, with interesting properties.^75^ Exciting photoluminescence behavior, optical stability, low toxicity, and biocompatibility have made them stand out among others.^76,77^ Primarily, synthesis of CQDs is achieved by two methodologies: top-down and bottom-up approaches.^78,79^ Smaller molecules aggregate together to form larger ones in a bottom-up approach, whereas larger molecules are broken down to form smaller counterparts in a top-down approach.^80^ The synthesis of CQDs has been explored using pomegranate peel *via* pyrolytic and hydrothermal methods at temperatures of 160 °C and 180 °C. The resulting material synthesized has also demonstrated concentration-dependent growth inhibition against *Staphylococcus aureus* (SA).^81^

Unlike other methods, which use harsh and harmful chemicals, CQDs can also be synthesized in an alternative green procedure that avoids them. Microwave pyrolysis is one such method employed for the synthesis of CQDs with waste lemon peels collected from a juice shop.^82^ The collected peels were first cleansed with DI water and subjected to carbonization in a muffle furnace at 200 °C for 2 hours. This resulted in a black powder, which was then heated under microwave irradiation for 5 minutes at 150 °C. After purification, the average particle size was measured to be up to 4.46 nm, and the quantum yield was calculated to be 49.5% by using the relative photoluminescence quantum yield equation. TEM was used to examine the morphology and the size of the nanostructures, which displayed spherical particles with monodispersity. Also, the hydrodynamic particle size was calculated to be approximately 52 nm using dynamic light scattering.^83^ Conversion of lignocellulosic materials into carbon nanomaterials has gained significant interest in recent years.^84^ These biomass resources are mainly comprised of hemicellulose, cellulose, and lignin, which are abundant in agricultural wastes.^34^ They possess a tremendous potential for biochar production as they are mainly composed of carbon, which in turn offers a renewable and cost-effective feedstock for carbon-based nanomaterials. One such research explored the use of moso bamboo waste to extract organosolv lignin, known for its structural purity. This extracted source was then subjected to a two-step hydrothermal method to successfully yield luminous CQDs. The resultant CQDs displayed a strong green fluorescence with the highest quantum yield of 17.7%.^85^

Finally, a wide range of waste materials, from plastics to agricultural and food scraps, can be effectively upcycled into various CNMs with diverse technological applications. The synthesis method plays a crucial role in determining the final product's characteristics, offering a versatile and sustainable approach to waste management.

## 3. Comparative advancements in waste-derived carbon nanomaterials

Recent work on waste-derived carbon nanomaterials shows clear progress compared to earlier methods, especially in the efficacy of the material production and how well their structures can be controlled. In the past, many waste precursors, whether plastic, agricultural, or food waste, depended on very high pyrolysis temperatures and yielded mixtures with uneven particle sizes and inconsistent carbon quality. Advancements such as catalyst-assisted pyrolysis,^86,87^ improved hydrothermal processing,^88^ and controlled microwave treatment^89,90^ operate at lower temperatures and produce more uniform materials. Manipulating heating profiles, pressure conditions, and precursor preparation have also made it easier to tune defect levels, pore structures, and dopant incorporation.^39,43,91^

These developments have had a direct impact on electrochemical sensing. Older waste-derived carbon electrodes usually showed moderate electron transfer behaviors and detection limits in a micromolar range.^92^ Electrochemical sensors produced over the past few years regularly achieve low nanomolar range or even sub-nanomolar detection limits.^93,94^ Collectively, these improvements demonstrate that waste-derived CNMs have matured from basic carbon alternatives to high-performance electrode materials comparable to conventionally synthesized carbon nanostructures.

## 4. Critical parameters influencing the synthesis and properties of WDCNMs

Carbon nanomaterials have become popular due to their exceptional electrical, mechanical, and thermal properties.^95–99^ These unique properties have been key in various fields such as solar cells, biosensors, fuel cells, drug delivery, bioimaging, *etc.*^100–104^ CNMs, like CNTs, graphene, and CQDs, have been explored by various researchers, but the precursor source of waste resources has been a new development.^105–109^ This addition of waste resources induces a range of properties in the materials.^110–112^ Synthesis of advanced nanomaterials such as CDs, porous carbon, and CNTs predominantly from waste materials employs the use of sustainable, waste-derived precursors and energy-efficient synthesis techniques^113–116^(Table 1). As discussed above, a wide range of waste resources like agricultural wastes, pharmaceutical wastes, plastic wastes, industrial by-products, *etc.*, have been used to generate carbon nanomaterials in the past few years^117–119^

**Table 1:** Summary of recent studies using different waste materials as precursors for carbon nanomaterial synthesis. The table highlights the type of waste, the conversion route employed, and the resulting carbon structures, demonstrating the wide range of carbon nanomaterials that can be produced from agricultural, industrial, municipal, and pharmaceutical wastes

Raw material used	Synthesis method	Derived carbon material	Reference
**Agricultural & lignocellulosic biomass**			
Cow dung	Carbonization followed by the hydrothermal method	Mg-doped carbon dots	152
Fruit waste	Slow pyrolysis followed by microwave-assisted synthesis using ferrocene catalyst	MWCNT	86
*Corchorus olitorius* (molokhia)	Microwave method (900 W for 10 min)	Nitrogen-doped carbon dots	134
Cellulose	Hydrothermal methodology (200 °C for 6 h)	Cellulose-derived carbon quantum dots	132
Sugarcane Bagasse pith	Pyrolysis with KOH activation	Multiscale carbon nanosheets	34

**Industrial & municipal waste**			
PET plastic (from water bottles)	Pyrolysis (815 °C) followed by Arc discharge method	Nano-channeled ultrafine carbon tubes	153
PET plastic	Carbonization with KOH treatment	Porous carbon	153
Iron tailings (as iron precursor)	Chemical acid leaching to extract iron, followed by co-precipitation	Fe_3_O_4_ magnetic nanoparticles	143

**Pharmaceutical waste**			
Expired ciprofloxacin tablets	Hydrothermal method (200 °C for 7 h)	Expired ciprofloxacin-derived carbon dots	131

### 4.1. Influence of temperature and pressure

The influence of temperature and pressure during the synthesis of these nanomaterials plays a vital role in tuning the mechanical and chemical properties of the desired nanomaterials.^120–122^ Hydrothermal synthesis often operates under high pressure in autoclaves at temperatures ranging from 180 to 300 °C for several hours.^123–125^ This pressurized aqueous environment is chosen due to the use of high-moisture feedstocks, which require extensive pre-drying.^126^ Under these conditions, the use of a pressurized atmosphere makes the conversion highly energy-efficient.^127^ Alternatively, the microwave synthesis is mostly valued for its speed, achieving conversions in ultra-short times, often in minutes or seconds.^128^ On the other hand, the pyrolysis methods utilize higher temperatures ranging approximately 300 to 1200 °C, performed under an inert atmosphere to prevent oxidation and control structural development.^129^ Along with pressure and temperature, how long or how short the reaction is carried out also alters the parameters of the materials.^130^

One such use of these parameters was explored with expired ciprofloxacin tablets to derive carbon dots successfully. The hydrothermal reaction was carried out for 7 h at 200 °C in an oven.^131^ In another study, textile-derived microplastic waste that is collected from washers and dryers was collected and subjected to a carbonization process in a batch reactor under a desired temperature of 200 °C, 250 °C, and 300 °C at a pressure of 22 bar, 40 bar, and 80 bar, respectively. The influence of different residence times was also investigated at 1 h, 4 h, and 8 h. This study revealed that the hydrogen content of the products decreased with increasing temperature, indicating a higher level of carbonization. At 250 °C in 4 h, the total solid carbon yield to amorphous carbon was observed to be 100% whereas at 300 °C for 4 h, the solid carbon yield to filamentous carbon was about 88%. The study concluded that a temperature of 250 °C and a residence time of 4 h can be ideal for plastic microfibers conversion, whereas a temperature of 300 °C at 4 h was found to be optimal to produce valuable carbon products like graphene and graphite.^72^ The influence of various temperatures and times might not be uniform for all the raw materials ever used. It varies from material to material based on their basic chemical and physical framework. CQDs derived from cellulose under hydrothermal methodology, leveraging a temperature of 200 °C for 6 h in a Teflon-lined autoclave reactor, successfully exhibited distinctive structural characteristics, expressing quasi-spherical morphology with an average diameter of approximately 7 nm and a band gap of 4 eV.^132^

### 4.2. Effect of time on WDCNMs

In the case of high-temperature usage, pyrolysis is the most common.^133^ A study explored the use of this method with fruit waste to derive multi-walled carbon nanotubes. This work explores the potential of 5 minutes microwave synthesis of MWCNTs from biochar at 100 °C. The fruit waste was first converted to biochar *via* a slow-pyrolysis method at different temperatures of 300 °C, 400 °C, and 500 °C at low heating rates and a ramping time of 5–30 minutes. The as-derived biochar is combined with ferrocene at a 1 : 1 ratio to subsequently yield CNTs with a diameter of 68 nm. The morphological and unique properties were confirmed by various characterization methods.^86^ The dependence of time on microwave irradiation method was studied when CDs were derived from *Corchorus olitorius* plants. Various time frames of 2, 4, 6, 8, 10, and 11 minutes were considered for the experiment. Similarly, to optimize the microwave power level, comparative analyses were done for 300, 450, 600, and 900 W, which led to the confirmation of a 900 W power supply for the experiment. Therefore, under an optimal condition of 10 minutes with a 900 W microwave supply, successful highest quantum yield was observed.^134^

### 4.3. Choice of solvents and reagents

Like the mechanical properties altered by the temperature and time, the choice of solvents and agents influences the chemical properties of the materials derived.^135–137^ In most cases, the distilled or deionized water is the primary choice of solvent for many hydrothermal or microwave-assisted syntheses.^138,139^ For metal-doped or porous carbon materials, chemical agents are essential for the surface porosity and morphology and also the electrochemical conductance of the material.^140–142^ Highly alkaline pH regulators like sodium hydroxide and ammonium hydroxide are used in co-precipitation reactions for magnetic nanomaterials to ensure the required rapid precipitation reaction of ferric and ferrous precursors.^143^ Strong acids like sulfuric acid and hydrochloric acid are also used as chemical activators or in pre-treatment steps to extract Fe^3+^ at 90 °C.^144^ Many biomass sources, like molokhia or Kentucky bluegrass, naturally contain nitrogen-rich biomolecules, enabling *in situ* doping during synthesis, which avoids the need for external heteroatom dopants and enhances catalytic activity.^145^ Precisely, the surface area, porous structure signify the electrochemical properties of waste-derived CNs. Chemical doping, thermal heating, as well as acidic treatments can adjust the position and functional groups of the nanomaterials. The doping of metallic and non-metallic elements like transition metals, p-block gases, and heavy metals usually alters the surface porosity and morphology of the nanomaterials.^142^ Nitrogen doping is employed because it enhances the stability and aqueous dispersion of CDs and provides active sites for analyte interactions and catalytic activity by modifying the carbon's electronegativity.^146^ Co-doping, such as with nitrogen and sulphur or nitrogen and oxygen, is often used to introduce a high density of defects and achieve a high specific surface area up to 1437.12 m^2^ g^−1^, which facilitates enhanced chemical adsorption and unique catalytic properties, often used to generate materials with multi-enzyme mimicking activities.^144^ For instance, doping was specifically used to generate biomimetic catalytic sites on porous carbon for improved electroanalytical applications.^147^ The deliberate incorporation of oxygen-containing functional groups (such as hydroxyl, carboxyl, and carbonyl) is essential for increasing hydrophilicity, establishing strong surface defect states, and facilitating electron transfer pathways beneficial for photocatalytic and nanosensing performance.^148^ In the case of magnetic and catalytic materials, metal doping/impregnation (*e.g.*, Fe, Mg) is used to create nanozymes exhibiting peroxidase or oxidase mimic activity, or to yield unique core–shell structures.^149^ Additionally, structural modification techniques like using molten salts are critical for guiding carbon morphology, suppressing oxidation, promoting high graphitization, and acting as porogens to control the development of hierarchically porous structures.^150,151^

## 5. Application of CNMs in electrochemical sensors

Electrochemical sensors have emerged as a promising tool for detection and quantifying different samples like biomolecules, contaminants which are immensely useful in diagnostics,^154^ environmental monitoring^155^ and food safety.^156^ These sensors are known for their advantages in sensitivity, selectivity, and miniaturisation potential for on-site monitoring.^157,158^ Incorporation of nanoparticles in sensors is crucial due to their ability to enhance electron transport and also to immobilise various components on the electrode surface.^159^ With the right catalysts and optimised conditions, electrochemical sensors have proven to detect samples at even picomolar ranges.^160^

Electrochemical sensors work by converting chemical information to a measurable electrical signal through reactions occurring at an electrode surface. When the target analyte comes into contact with the sensor, it undergoes either oxidation or reduction, generating a flow of electrons that produces a current, changes potential or alters impedance, depending on the sensing mode.^161^ These changes are often directly proportional to the concentration of the analyte, allowing for highly sensitive and selective detection.^162^ The performance of a sensor is also largely influenced by the electrode material, surface modifications, and the kinetics of electron transfer occurring at the interface.^163^ As the sensors function under relatively mild conditions and respond rapidly to changes, they have become indispensable tools for monitoring biological molecules, environmental contaminants, and industrial processes.^164,165^

Using waste-derived carbon nanomaterials as catalysts in these sensors represents a highly promising and impactful pathway in sensor design. These materials combine sustainability with strong electrochemical performance.^166^ The waste-derived carbon nanomaterials have a lot of variations in their electrochemical properties due to their differences in specific surface area and morphological behaviour, while most of the structural properties of CNMs vary along with their structural framework of the raw materials chosen for synthesis.^167^ The morphological expansion and the changes in their carbonic framework result in variation of electronic conduction, which is an appropriate reason behind the difference in the qualitative and conductometric behaviour of various carbon nanomaterials.^168^

### 5.1. Detection of pharmaceutical pollutants

**Fig. 4:**
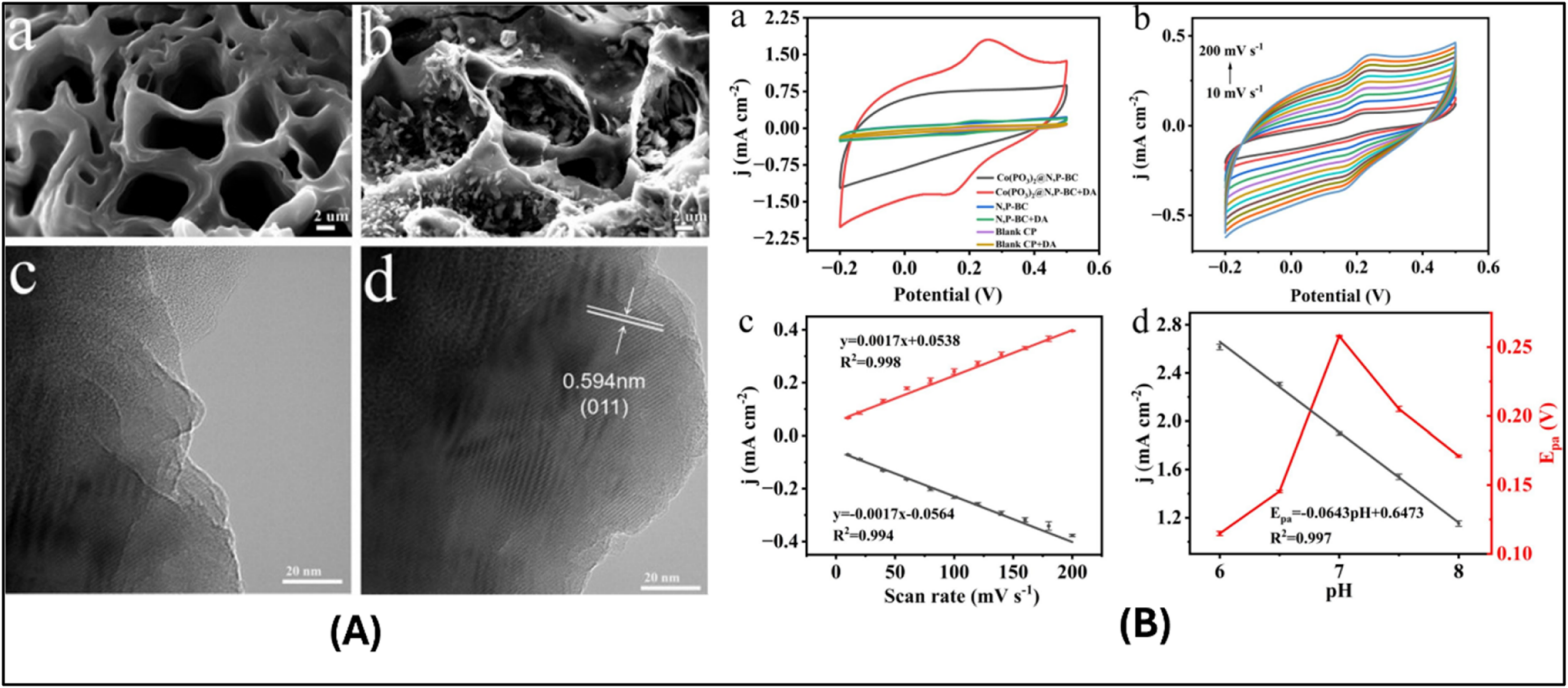
(A) (a and b) Scanning electron microscopy images of N,P-BC and Co(PO_3_)_2_@N,P-BC. (c and d) HRTEM images of Co(PO_3_)_2_@N,P-BC. (B) (a) Cyclic voltammograms of bare carbon paper, N,P-BC/CP, and Co(PO_3_)_2_@N,P-BC/CP in 0.1 M PBS (pH 7) with and without 100 µM DA, (b) CVs of Co(PO_3_)_2_@N,P-BC/CP at varying scan rates (10–200 mV s^−1^) in the presence of 50 µM DA, (c) Corresponding plots of current density *versus* scan rate, and (d) Variation of *I*_pa_ and *E*_pa_ with pH. Reproduced from ref. 172 with permission from Elsevier B.V., copyright 2025.^172^

A study developed with a molecularly imprinted electrochemical sensor uses Asphalt-derived carbon (ADC) and silver nanoparticles (AgNPs) to modify a glassy carbon electrode (GCE). The sensing platform fabrication involved immobilizing ADC on the GCE to provide a large specific surface area and improved structural stability. AgNPs were electrodeposited onto the ADC/GCE to optimize electron transfer kinetics and amplify the electrochemical signal. This sensor is designed for the sensitive detection of Ribavirin (RIB) in poultry feed samples. Under optimized differential pulse voltammetry (DPV) conditions, the sensor exhibited a wide linear range from 1 to 500 ng mL^−1^. It achieved a low detection limit (LOD) of 0.4 ng mL^−1^. The overall performance evaluation demonstrated outstanding sensor repeatability and long-term stability, confirming its reliability.^169^ Another sustainable electrochemical sensor utilizes a cobalt sulfide/reduced graphene oxide (CoS/rGO) nanocomposite. The synthesis employed a straightforward, low-cost method integrating thiourea and cobalt acetate to form CoS, with the rGO derived from recycled plastic waste. The optimized formulation contained 40 wt% rGO, which significantly enhanced electrical conductivity and surface activity. The composite was evaluated for the detection of paracetamol (PCT). The optimized CoS/rGO composite achieved a sensitivity of 12.4 µA mM^−1^ cm^−2^. It demonstrated a LOD of 0.2 µM, which is approximately 7.5 times lower than that of pristine CoS. The linear range observed was 0–5 mM. The sensor exhibited excellent operational stability over 100 consecutive cycles and high repeatability with a relative standard deviation (RSD) below 2.5%.^170^ Rice husk-derived graphene (RHG) was used to modify a carbon paste electrode (CPE) and used for the electrochemical detection of mefenamic acid (MA), in the presence of cetyl-trimethylammonium bromide (CTAB), a cationic surfactant that improves the electron transfer kinetics and surface morphology. Using square wave voltammetry (SWV), this sensor achieved a detection limit of 2.13 nM. The sensor demonstrated high sensitivity and selectivity in detecting MA. The study confirms that the presence of metal ions and other interfering elements does not disrupt the MA reaction, making it suitable for analysis in human urine, blood serum, and breast milk samples.^171^ Biochar derived from waste pomelo peel was co-doped with nitrogen and phosphorus (N, P-BC) along with cobalt metaphosphate (Co(PO_3_)_2_), and used as an electrode material in CPE (Fig. 4A & B). (Co(PO_3_)_2_)@N, P-BC/CPE sensor was developed for dopamine detection. It showed a low LOD of 0.547 µM. The sensitivity of this sensor was examined in the presence of uric acid (UA), urea, potassium chloride (KCl), ascorbic acid (AA), glucose (Glu), and glycine (Gly).^172^

Another sensor platform that uses a dual biomass-derived porous carbon, synthesized from psyllium husk (PSH) and eggshell powders (ES), where the eggshell powder acts as an *in situ* template and as an activating agent. These molecularly imprinted polymers are constructed using dimetridazole (DMZ) as a template and *o*-phenylenediamine (oPD) as the functional monomer assembles on a gold electrode. The LOD was experimentally identified to be 1.8 nM and demonstrated excellent sensitivity. The selectivity of the sensor was challenged with twice the concentration of structurally related compounds and ten times the concentration of metal ions.^173^ Another interesting research that used microbial metabolites such as short-chain fatty acids, phenolic compounds, and indole derivatives in biological waste samples. This sensor platform utilizes carbon nanodots (CNDs), which are synthesized from citric acid as a precursor through hydrothermal processes. The as-synthesized CNDs were used as catalysts on GCE to form the modified sensor. The LOD for the sensor was experimentally found to be 0.128 µM. The CND sensor exhibited high efficiency in countering interference, achieving selectivity percentages ranging from 71.3% to 96.8%. The sensors displayed high reproducibility (RSD < 4.21%) and maintained excellent stability, preserving over 90% of the signal response after ten successive cycles.^174^

### 5.2. Detection of biomolecules and clinical analytes

Discarded waste pistachio shells, which are collected from a local market in new Taipei city, were thoroughly washed in DI water to eliminate impurities, followed by mechanical grain mill grinder at 28 000 rpm for 5 minutes, which resulted in a light brown powder. It was then processed through hydrothermal carbonization as hydrochar carbon (HC), which noted for having carbonyl and carboxyl functional groups on their surface. Incorporation of processed shells along with citric acid in the stirrer provided acidic carbonization along with stability to prevent strong agglomeration. This HC is modified onto a Screen-Printed Carbon Electrode (SPCE) to successfully detect glucose and lactate in human sweat samples using DPV techniques. Low LOD for glucose (0.28 µM) and for lactate (0.37 µM) were observed. The sensor exhibited excellent sensitivity, reproducibility, and stability over a period of 15 days. The DPV analysis exhibited negligible interference for the simultaneous detection of glucose and lactate.^175^ Food waste is rich in organic compounds and can be used to synthesise carbon. Similarly, Chinese cabbage leaves that are copper-enriched are synthesised by soaking the waste in different copper salt solutions, followed by pyrolysis. This sensor successfully detects ascorbic acid with a high sensitivity, and a linear response range was observed from 0.2 µM to 7 mM.^176^

### 5.3. Detection of heavy metal ions

A recent study demonstrated the use of *Parthenium hysterophorus* biomass as a sustainable precursor for fabricating a heavy-metal electrochemical sensor. The plant waste was converted into a graphitized carbon material through catalytic graphitization, producing a porous, graphene-like nanostructure with high crystallinity and uniformly distributed silver nanoparticles. This waste-derived nanocomposite served as an efficient sensing platform for Pb^2+^ and Hg^2+^ detection, with cyclic voltammetry and differential pulse anodic stripping voltammetry (DPASV) confirming sensitive and selective responses. The reported detection limits were 600 nM for Pb^2+^ and 300 nM for Hg^2+^, with good reproducibility (RSD ∼3.2%) and stable performance over extended storage. Real-sample testing in tap water further demonstrated reliable Pb^2+^ detection at ∼400 nM, highlighting the potential of biomass-derived carbon nanomaterials for practical heavy-metal monitoring.^94^ Overall, the use of waste-derived carbon nanomaterials has proven highly effective for electrochemical detection of heavy metal ions, offering a sustainable alternative to conventional carbon sources.

The above analysis confirmed that modern electrochemical sensors are profoundly defined by the principles of sustainability and high analytical precision, successfully leveraging the valorization of diverse biowaste and recycled materials to create advanced sensor interfaces.^177^ These specialized carbon and composite platforms achieve exceptional electrochemical performance across a broad spectrum of targets, including ultra-low detection limits (Table 2). The demonstration of high selectivity and robust anti-interference capacity allows reliable quantification of analytes in complex media such as human serum, environmental water, and poultry feed samples.^178^ While future efforts must focus on improving large-scale synthesis, addressing batch variability, and substituting environmentally critical reagents, the current success validates a pathway for cost-effective, highly reproducible, and reliable sensing technologies.^179^ Ultimately, the integration of these sophisticated, waste-derived sensors into portable, miniaturized, and intelligent systems is poised to advance real-time monitoring solutions in both environmental protection and clinical diagnostics.^180^

**Table 2:** Overview of recent studies utilizing waste-derived precursors for the synthesis of carbon-based nanomaterials applied in electrochemical sensing. The table summarizes the synthesis approaches, electrochemical techniques, target analytes, and LOD of the sensors discussed

Raw material	Synthesized nanomaterial	Synthesis method	Electrochemical technique	Target analyte	LOD	Reference
Rice husk	Graphene (RHG)	Chemical activation	SWV	Mefenamic acid	2.13 nM	171
Psyllium husk & eggshell	Biomass-derived porous carbon	Pyrolysis & hydrothermal process	DPV	DMZ	1.8 nM	173
Coffee grounds	Cu_2_O_3_-carbon quantum dots/graphene nanoribbons	Hydrothermal method & ultrasonication	DPV	PCT	0.016 µM	181
Pistachio shells	HC	Hydrothermal carbonization (HTC)	DPV	Glucose	0.28 µM	175
Pistachio shells	HC	HTC	DPV	Lactate	0.37 µM	175
Chinese cabbage leaves	Copper-enriched carbonaceous material	Pyrolysis	Cyclic voltammetry (CV)	AA	0.05 µM	176
Eggshells	WO_3_/Cu-doped CaO nanocomposite	Calcination	SWV	Epinephrine (EPI)	0.00246 µM	182

## 6. Future outlook

The future for waste-derived carbon nanomaterials in electrochemical sensing looks very promising. As synthesis methods continue to improve, researchers are gaining control over features like pore size, surface chemistry, defect levels, and heteroatom doping.^183^ This means that carbon materials made from agricultural or plastic waste can increasingly compete and, in some cases, outperform traditional nanocarbons in terms of catalytic activity and sensing efficiency.^184^ Newer, greener production approaches, such as plasma activation, microwave-assisted carbonization, and low-energy hydrothermal processing, will make these materials even more scalable and environmentally friendly.^185^ With the enormous amount of waste generated globally every day, the use of waste materials as a potential precursor for the synthesis of high-value carbon nanomaterials might also help in terms of waste management, when considering mass production.^186^

Looking ahead, waste-derived carbons are expected to play a major role in the development of wearable, implantable, and real-time monitoring sensors for health and environmental applications. Their low cost and abundant availability make them ideal candidates for disposable or portable sensing devices, especially in settings where affordability is critical.^187^ At the same time, combining these carbons with metals, MXenes, conducting polymers, or biomolecules will open the door to high-performance hybrid sensing platforms with improved sensitivity, selectivity, and resistance to fouling.^117^ To fully realize this potential, future research must focus on standardizing preparation methods and understanding how structural features translate to sensing performance. With continued progress, waste-derived carbon nanomaterials are well-positioned to become essential components of the next generation of sustainable and high-performance electrochemical sensors.^188^

Furthermore, a deeper understanding of the long-term stability and biocompatibility, and ecotoxicity profiles of waste-derived CNMs is essential for human health and environmental safety.^189^ Future efforts must prioritize creating multi-functional CNM composites and conducting rigorous *in vivo* studies and full lifecycle assessments.^190^ Ultimately, the successful transition of waste-derived CNM technology from the lab to the market will not only drive the development of high-performance sensing and energy systems but also fundamentally reinforce the principles of the circular economy, positioning waste as a critical resource for next-generation technology.

## 7. Conclusion

The intersection between global waste management crises and the urgent need for sustainable material innovation is precisely where waste-derived carbon nanomaterials offer a revolutionary pathway. This review underscored the profound potential of upcycling diverse waste streams ranging from plastic and electronic waste to agricultural biomass into high-value CNMs such as CNTs, graphene, and CQDs. We detailed that the material's final structural, morphological, and surface properties, which are critical to performance, are precisely controlled by tuning synthesis parameters, including pyrolysis temperature, hydrothermal conditions, and chemical doping, to yield specific 0D, 1D, and 2D nanostructures. This versatility fundamentally redefines waste resources not as disposal challenges, but as essential, untapped reservoirs of advanced carbon precursors.

The direct impact of these sustainable CNMs is particularly evident in the domains of electrochemical sensing technologies. In sensing, waste-derived CNMs have enabled the development of highly sensitive, selective, and robust sensor interfaces, achieving ultra-low detection limits for analytes across environmental, clinical, and food safety applications. Their high conductivity, large specific surface area, and customizable defect sites significantly enhanced electron transfer kinetics, validating their role as superior electrode modifiers.

## Author contributions

S. V.: writing the original draft, formal analysis, methodology, data curation, and conceptualization. A. K. S.; writing, editing, supervision, resources, visualization, validation, resources, project administration, and funding acquisition.

## Conflicts of interest

The authors declare that they have no known competing financial interests.

## Data Availability

All experimental data discussed in this work were obtained from previously published articles, which are appropriately cited throughout the manuscript. No additional datasets were used or excluded.
